# Atomic Force Microscopy Is a Potent Technique to Study Eosinophil Activation

**DOI:** 10.3389/fphys.2019.01261

**Published:** 2019-10-01

**Authors:** Peter Eaton, Constança Pais do Amaral, Shirley C. P. Couto, Mariangela S. Oliveira, Andreanne G. Vasconcelos, Tatiana K. S. Borges, Selma A. S. Kückelhaus, José Roberto S. A. Leite, Maria Imaculada Muniz-Junqueira

**Affiliations:** ^1^Instituto de Medicina Molecular, Faculdade de Medicina, Universidade de Lisboa, Lisbon, Portugal; ^2^LAQV-REQUIMTE, Department of Chemistry and Biochemistry, Faculty of Sciences, University of Porto, Porto, Portugal; ^3^Laboratory of Cellular Immunology, Pathology Area, Faculty of Medicine, University of Brasília, Brasília, Brazil; ^4^Research Center in Morphology and Applied Immunology, Morphology Area, Faculty of Medicine, University of Brasília, Brasília, Brazil

**Keywords:** atomic force microscopy, acute asthma, eosinophils, cell morphological changes, allergic diseases, advanced microscopy

## Abstract

Eosinophils are multifunctional cells with several functions both in healthy individuals, and those with several diseases. Increased number and morphological changes in eosinophils have been correlated with the severity of an acute asthma exacerbation. We measured eosinophils obtained from healthy controls and individuals with acute asthma using atomic force microscopy (AFM). In the control samples, cells showed more rounded morphologies with some spreading, while activated cells from symptomatic individuals were spreading, and presenting emission of multiple pseudopods. Eosinophils presenting separate granules close to the cells suggesting some degranulation was also increased in asthma samples. In comparison to histopathological techniques based on brightfield microscopy, AFM showed considerably more details of these morphological changes, making the technique much more sensitive to detect eosinophil morphological changes that indicate functional alteration of this cell. AFM could be an important tool to evaluate diseases with alterations in eosinophil functions.

## Introduction

Eosinophils are multifunctional cells that express an array of proinflammatory cytokines, chemokines, and lipid mediators, express receptors for complement, immunoglobulins, and cytokines, and have been implicated as important participants in the innate and adaptive immune responses (Liao et al., 2016). Studies investigating the roles of eosinophils in both healthy and diseased individuals demonstrate that the activities of these granulocytes are complex and wide-ranging. They contain several mediators necessary to regulate both innate and adaptive immune responses (Jacobsen et al., 2012; Weller and Spencer, 2017). Numerous studies show a role for the eosinophil in disease, including allergic diseases, such as asthma, atopic dermatitis, chronic urticaria, chronic eosinophilic pneumonia, and the hypereosinophilic syndrome (Gleich, 2000), skin diseases (Long et al., 2016), eosinophilic esophagitis (Cianferoni and Spergel, 2016; Botan et al., 2017) and gastrointestinal disorders (Uppal et al., 2016). Furthermore, they are also implicated in host defense against helminth infections and as a cause of inflammation and tissue damage in parasitic, viral, fungal, and bacterial infections (Ravin and Loy, 2016), and there is evidence for the cytotoxic effect of eosinophils on tumor cells (Davis and Rothenberg, 2014). They are also implicated in several other diseases (Jacobsen et al., 2012).

Eosinophils are implicated in pathophysiology of asthma that is a chronic inflammatory disease of the airways currently affecting some 235 million people, and is more common among children than in adults (World Health Organization [WHO], 2018). From an immunological perspective, asthma is a heterogeneous disease marked by a disorder in T helper type 2 (Th2) cells in the lung, along with the presence of a large number of eosinophils in the airways. In some patients a predominantly neutrophilic inflammation, controlled by the Th17 subset of T helper cells, or in some cases by eosinophilic inflammation controlled by type 2 innate lymphoid cells (ILC2 cells) acting together with basophils may occur (Lambrecht and Hammad, 2015).

The increased number of eosinophils in the peripheral blood from patients with asthma is frequently associated with bronchial hyper-responsiveness (Fahy, 2009; Tabatabaian et al., 2017). These cells suffer metabolic, functional, and phenotypic alterations when activated, and secrete diverse inflammatory substances, including cytokines, lipid mediators, and toxic granule proteins (Kita, 2011; Muniz-Junqueira et al., 2013).

Functional properties of eosinophils can be divided into at least three stages including resting, partial activation, and full activation (Kita, 2011) that shown correspondence with its morphological characteristic (Muniz-Junqueira et al., 2013). Recently, Muniz-Junqueira et al. (2013) demonstrated that the state of activation of eosinophils could be a viable, reliable and objective marker for acute exacerbations of the disease, distinguishing it from asymptomatic asthma. For this purpose, the quantification of eosinophils with morphological changes was carried out by optical microscopy, using histopathological staining.

**FIGURE 1:**
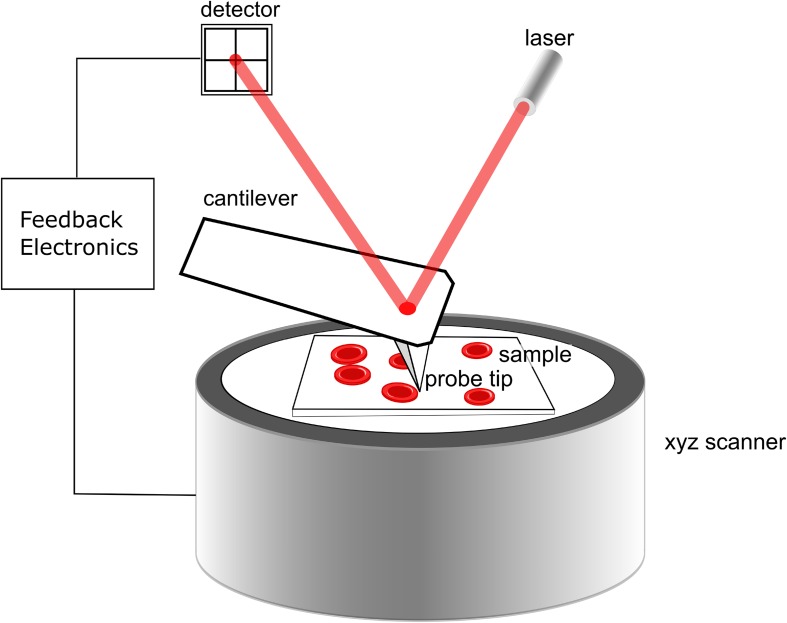
Schematic of the operation of the AFM. The xyz scanner raster scans the sample under the sharp probe. Interactions of the probe with the sample cause the cantilever to deflect (or to damp vibration in tapping mode). This is detected by the photodetector and feedback electronics ensure the interaction force between probe and sample are kept minimal.

Atomic force microscopy (AFM) is a technique used to view and measure surface structures with high resolution and accuracy. AFM uses a physical probe to scan over a sample surface, while maintaining a very small force between probe and sample, as illustrated in Figure 1. AFM is particularly appropriate for biological samples, since it requires no special environmental conditions, and can image soft materials such as cells without fixing or drying (Morris et al., 2009; Eaton and West, 2010). A very wide variety of cells have been probed by AFM, including macrophages (Bitler et al., 2012), thyroid cells (Prabhune et al., 2012), and hepatocytes (Eaton et al., 2012). Unlike electron microscopy, AFM can examine cells in ambient conditions, not requiring the use of vacuum. Avoiding full dehydration of cells can allow AFM to probe structures without dehydration, which can lead to morphological alterations, even with fixation. In addition, AFM is extremely surface sensitive, able to detect sub-nanometer morphological features with ease. Taking into consideration that morphological characteristics of eosinophils activation can be used as a marker of severity of asthma exacerbation and that AFM may be more sensitive to morphological features of cells than optical microscopy, we hypothesized that AFM could better shown the morphological changes in eosinophils than optical microscopy. Thus, evaluation of eosinophil using an AFM could be an important tool in research on functions of these cells in healthy and in several diseases, and could improve the understanding of its function in several diseases. In the current work, we apply AFM to evaluate the morphological aspects of eosinophils from asthmatic individuals in comparison with the same eosinophil evaluated by optical microscopy. We show that using this method, it’s possible to obtain much more details of morphological aspects of eosinophil activation than that observed by optical microscopy.

## Materials and Methods

Eosinophils were obtained from peripheral blood (1 ml) from 3 asthmatic individuals and 3 healthy controls upon approval from the Ethics Committee of the Faculty of Medicine, University of Brasília (process number 038/1), and all volunteers gave written informed consent for their participation in the study. Collection was made according to the methodology from Muniz-Junqueira et al. (2013).

The eosinophils were obtained from healthy controls and asthmatic individuals by venipuncture. Samples of 40 μL whole blood collected without anticoagulant were placed on each field of microscope slides, which were marked with a 7 mm diameter round field using oil ink mixed with epoxy resin, to allow adherence to glass, repeated in eight wells for each sample. After incubation at 37°C for 45 min in a humid chamber, slides were washed with a 0.15 M phosphate-buffered saline (PBS; pH 7.2) at 37°C to eliminate non-adhered cells, fixed with methanol, stained with 10% Giemsa for 10 min, washed with water and finally air-dried. Eosinophils that remained adhered to glass slide were evaluated by optical microscopy and AFM. This method of separation of phagocytes by adherence to slide has long been used (Jacques and Bainton, 1978; Grinnell and Feld, 1981; Murphy-Ullrich and Mosher, 1985; Muniz-Junqueira et al., 2003, 2013).

For previous standardization of the test, eosinophils from whole blood collected without anticoagulant or collected with heparinized tubes were tested. The results showed no difference between adherence to slide and characteristics of normal or activated eosinophils by optical microscopy. After adherence to slide for 45 min, eosinophils from healthy individuals showed insignificant characteristics of activation (Muniz-Junqueira et al., 2013). Similarly, no different results were observed whether venous or arterial blood was evaluated.

Eosinophils were separated from whole blood by the adherence to glass slide, and cells remained adhered on glass slide in a proportion similar to that observed in the peripheral collected blood. For healthy individuals, 12,534 ± 5,050 cells/marked area remained adhered on glass slide (93.5 ± 1.08% neutrophils, 5.63 ± 0.85% monocytes, and 0.87 ± 0.63% eosinophils). Thus, higher than 85% of eosinophils/mm^3^ in sample of circulating blood remained adhered to slide. The percent of adhered cells on the slide was similar to that observed by Jacques and Bainton (1978). There was correlation between eosinophils quantified in peripheral blood and that adhered on glass slide. The median number of eosinophils in peripheral blood was 245/mm^3^ for healthy individuals and 635/mm^3^ for asthmatic individuals.

The cells were initially identified by optical microscopy and subsequently the same (Giemsa stained) cells were studied by AFM. The morphology was assessed using either a TT-AFM instrument (AFMWorkshop, United States) in vibrating mode in air using cantilevers with approximately 300 kHz resonant frequency (AppNano, United States), or a Nanowizard 4 instrument (JPK, Germany) in AC mode using the same cantilevers. There was no difference noted between the results from the two AFM instruments. Using both AFM and optical microscopy, we looked for the presence of single or multiple pseudopods, granule release and spreading. Optical images were obtained use a 100× oil immersion objective, and images were optimized for contrast.

## Results

**FIGURE 2:**
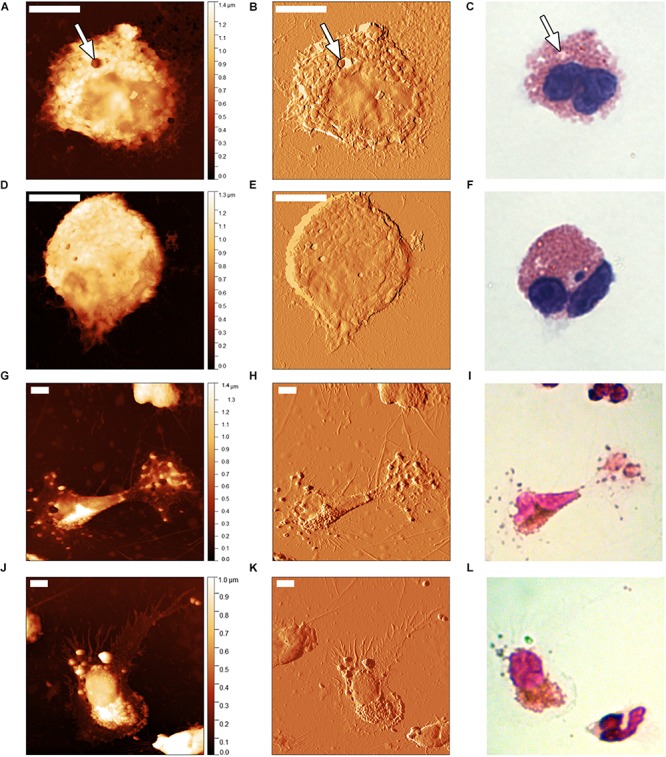
Atomic force microscopy images showing the morphological changes in activated eosinophils from individuals with acute asthma **(G–L)** in relation to normal eosinophils from healthy individuals **(A–F)**. First column – height image; second column – amplitude image; third column – optical microscopy images. All scale bars represent 5 μm. Arrows indicate features discussed in the text.

Representative images illustrating results of the evaluation of morphological aspects of eosinophils by optical microscopy and AFM are shown in Figure 2. In the control sample, optical microscopy shows the key characteristics of eosinophils: two-lobe nucleus and numerous cytoplasmic granules (Figures 2C,F). By AFM, the cell morphology appeared slightly rounded in shape, with well-defined contour and a rough surface, mainly in the cytoplasm where there are granules (Figures 2A,B,D,E). The nucleus with two lobes is visible in the height image (Figure 2A). Not all cells displayed a clear, two-lobed nucleus in AFM, presumably because it was overlaid by the rest of the cell. The amplitude image (Figures 2B,E) highlights the roughness of the surface. There are features which could represent granules, but few, if any pseudopods visible. It’s also worth mentioning that in Figures 2C,F some globular features (arrow) appear more lightly colored, and they are also shown in AFM images as depressions in the height images (Figures 2A,B,D,E) (arrow).

In the symptomatic asthmatic individual samples, many highly activated eosinophils were found, presenting irregular, spreading morphology, emission of multiple pseudopods and the presence of separate granules close to the cells suggesting likely some degranulation (Figures 2G–L). Note the loss of defined contour and of rounded aspect, distinctive of activated eosinophils. It should also be noted that in one cell, we counted 55 pseudopods, a number much higher than that observed by optical microscopy. It’s worth mentioning that although typical images are shown here, in samples from asymptomatic healthy individuals, a small proportion of activated eosinophils were also seen. It’s also worth mentioning that in Figure 2C some globular features (arrow) appeared more brightly colored, and they are also shown by AFM images (Figures 2A,B) (arrow).

Table 1 shows data of quantification in 50 eosinophils from healthy control individuals and 50 eosinophils from the asthmatic individuals of the number of cells that were presenting the morphological features appearance of rounded or spread shapes, emission single pseudopod, 2–4 pseudopods, >4 pseudopods or showing signs of likely degranulation. The number of pseudopods in the asthmatic individual samples was high (Table 1) and they varied considerably in size. Pseudopods optically visible were 50–150 nm in diameter while very small ones were as small as 8 nm in diameter, many of which were invisible in optical microscopy. It is important to point out that the presence of some granules found in the healthy control (Table 1) could be related to sample preparation, since the manipulation of the sample can partially activate the cells. Cells presenting multiple (>4) pseudopods, were also seen very rarely in the control samples (Table 1).

**TABLE 1:** Number (percentage) of eosinophils with morphological changes characteristic of activation observed using atomic force microscopy in healthy control asymptomatic individuals versus symptomatic asthmatic individuals.

	**Asymptomatic healthy control *n* (%)**	**Symptomatic asthmatic individuals *n* (%)**
Rounded	48 (96)	18 (36)
Spread	2 (4)	32 (64)
Single pseudopod	0 (0)	3 (6)
2–4 pseudopods	3 (6)	5 (10)
>4 Pseudopods	1 (2)	43 (86)
Likely degranulating	12 (24)	40 (80)

*In both cases, the number of cells counted (*n*) was 50.*

## Discussion

Eosinophils are multifunctional leukocytes resident in various organs such as the gastrointestinal tract, mammary glands, and bone marrow, which play a part in the immune homeostasis of these organs, modulation of adaptive and innate immune responses, and host protection (Kita, 2011). In the human blood, these cells form heterogeneous populations with various magnitudes of activation regulated by the immunological environment (Kita, 2011).

It is known that eosinophil recruitment and presence of granular proteins such as major basic protein, eosinophil cationic protein, or eosinophil peroxidase are biomarkers for monitoring allergic inflammatory disorders, such as asthma (MacPherson et al., 2001). However, the precise knowledge on pathophysiological features of activated eosinophils has still been subject of extensive investigation. MacPherson et al. (2001) discuss how eosinophils are unique in their capacity to generate oxidants in severe asthma, being endowed with numerous basic and cytotoxic granule proteins that are released upon activation.

Additionally, considering the association between activation state of blood eosinophils and symptomatology of esophageal inflammatory disease, Botan et al. (2017) demonstrate that morphological changes in the cells were significantly higher in individuals with eosinophilic esophagitis than in the control group. Among the alterations described are spread cells, pseudopods, cytoplasmic vacuoles, moderate, and large granule release, and clusters of free eosinophil granules, as analyzed by optical microscopy.

Similarly, the study of morphological changes in blood eosinophils in acute asthma exacerbation in children performed by Muniz-Junqueira et al. (2013) showed a higher proportion of activated blood eosinophils in asthmatic children than in the control group, presenting emissions of multiple pseudopods, presence of cytoplasmic vacuoles, spreading, and presence of a cluster of free eosinophil granules.

In order to quantify the difference in images from optical microscopy and AFM observed by us in acute asthma and control individuals, and also to compare our data with those of Muniz-Junqueira et al. (2013), we counted the number of pseudopods emitted in cells displaying multiple pseudopods observed in this work. It is noticeable from our work that the number of pseudopods observed for each activated eosinophil was very high, when compared to that of the work of Muniz-Junqueira et al. (2013). For example, in a unique cell, we counted 55 pseudopods by AFM, whereas no more than seven pseudopods were visible in one eosinophil in the work of Muniz-Junqueira et al. (2013). This can be seen by comparing the number of pseudopods in the same eosinophils analyzed by AFM in Figures 2H,K in comparison with the same eosinophils seen by optical microscopy (Figures 2I,L). This suggests that the number of pseudopods and the detail of each are easier to observe in AFM than in traditional optical microscopy. This could be due to the ability of this high-resolution technique to detect smaller pseudopods, which would be optically invisible by optical microscopy. The images in the first and second columns of Figure 2, the height and amplitude images, were obtained simultaneously by the AFM. The height images are almost always shown in AFM images since they represent true three-dimensional topographic data, indicated by the height values next to the color scales. On the other hands, the amplitude images enable clearer visualization of both whole cells, and small features such as pseudopods (see, for example, Figure 2H) (Eaton and West, 2010).

Figures 2C,F of normal eosinophils stained by Giemsa and captured by optical microscopy show the presence of some globular features (arrow) that are much lighter colored than the cell body, and they are also shown by AFM images and depressions of “holes” (Figures 2A,B,D,E) (arrow). The explanation of these features is not fully clear. However, it is possible that these represent evidence of the degranulation process, and the brightly colored region image could be a reversal in granule core staining. There are several mechanisms for eosinophil degranulation. (1) Classical exocytosis of granules, by fusion of the membrane granule with the plasma membrane lipid bilayer. (2) Compound exocytosis, when a number of granules coalesce and fuse and the cationic protein content is released by a pore at the plasma membrane. (3) Piecemeal degranulation, where secretory vesicles bud from granules and may capture granule matrix and/or core content. (4) Cytolysis by eosinophil necrosis and disruption of eosinophil plasma membrane (Acharya and Ackerman, 2014). In piecemeal degranulation, granules may exhibit different degrees of empting of their contents dependent on the causative stimulus. It has been shown that the major basic protein localizes mostly in the crystalloid core of granules and eosinophil cationic protein and eosinophil-derived neurotoxin reside mainly in the granule matrix, leading to the classical image of the granule with a dense core and matrix. After degranulation, when there is loss of the usual granule core density, it appears that the major basic protein was released and they may show reversal of granule core staining (Peters et al., 1986; Saffari et al., 2014). In addition, since the methanol used in cell fixation stiffens the cell structure, dissolves lipids and coagulates proteins from cell membranes, it is possible that the pores resulting from the exocytosis are more evident in these fixed cells (Ganjali and Ganjali, 2013).

Eosinophils can show three stages: resting, partial activation, and full activation depending on stimuli they are subjected to (Kita, 2011). Eosinophil activation occurs generally in a highly complex molecular milieu, to which both plasma-derived proteins and cell-derived mediators may contribute (ErjefäLt and Persson, 2000; Kita, 2011). As early as in 1880, Berkart depicted activated eosinophils in fresh sputum from an asthma patient, and in 1892, Schmidt observed eosinophils and free eosinophil granules in sputum samples, as cited by Persson and Erjefalt (1997). In peripheral blood, hypodense eosinophils have consistently been described in asthmatic individuals and represent activated cells (Kroegel et al., 1994). The changes in light-density eosinophils include the appearance of cytoplasmic vacuoles, an alteration of the cell size and shape, an increase in the granule size, and these cells may be degranulated, contain less granules, and have an increased cell volume (Kroegel et al., 1994; Neves and Weller, 2009). The morphological aspects of eosinophil activation have already been evaluated using light (Pronk-Admiraal et al., 2002), confocal (Lacy et al., 2011) and electronic microscopy (ErjefäLt and Persson, 2000; Melo et al., 2005; Weller and Spencer, 2017). The technique used in our study permitted the eosinophils to adhere to the glass slide before evaluating of their activation. Our data suggest that the glass adherence may reveal the state of activation-associated morphology of these eosinophils. This methodology appears to be a more reliable indication of what will happen when these cells migrate to target tissue, such as pulmonary tissue in asthmatic patients or esophageal tissue in eosinophilic esophagitis patients (Muniz-Junqueira et al., 2013; Botan et al., 2017).

A limitation of this work was the small number of individuals studied. However, this can be justified because the aim of this work was only to compare the images observed using the AFM and optical microscopy. Another limitation of the work is that we do not know what is happening during the 45 min of incubation of whole blood to allow eosinophils to adhere to glass, and which are the factors determining the morphological changes observed in eosinophils in allergic patients. In addition, adherence to glass slide may activate adhered cells. However, the analysis of the activation state of eosinophils from the control group should minimize this limitation. Eosinophils of healthy control individuals have shown minimal morphological changes in eosinophils. However, it is not possible to fully rule out that some eosinophils were activated only because the adherence to glass. Therefore, we propose that this technique could be useful to determine the morphological aspects and mainly the activation state of eosinophils in several diseases, as we observed in asthma sufferers, and thus, might be useful as an improved method for better understanding the eosinophil morphology and function in several diseases, as we observed in asthma patients.

Literature reports studies of morphological aspects of eosinophil activation using transmission electron microscopy (TEM) and fluorescence microscopy, aiming to evaluate specific ultrastructural features of tissue eosinophil (Justinich et al., 1997; ErjefäLt and Persson, 2000). For Deng et al. (2010), although TEM has very spatial resolution and unlike AFM enables the analysis of intracellular components, a very involved and complex sample preparation means high potential for morphological alteration during processing.

On the other hand, AFM has great promise for high-resolution imaging and offers advantages over other technologies, including SEM, enabling imaging without full dehydration, applications in cellular structure characterization, physical property measurements locally and globally on a single cell, and 3D characterization applied in cellular research (Deng et al., 2010).

The method of eosinophil separation of the whole blood by glass adherence is a simple operation leading to a result that could serve as a biomarker. In comparison with other technique of cell separation, the technique described in this work is the one that causes less stress to the cells in the processing to obtain the eosinophils, since the blood obtained is directly placed immediately on the slide, without undergoing any other processing such as centrifugation in separation media or treatment with antibodies. Furthermore, for AFM analyses, no special procedure of the sample is necessary in comparison to that necessary for analysis by electronic microscopy, or confocal microscopy.

As a perspective, AFM could be an important tool to evaluate activation state of eosinophil and eosinophil extracellular traps in extracts, from, or directly in pulmonary tissues of asthmatic patients and esophageal tissue in esophagitis eosinophilic patients (Marturano et al., 2013; Saffari et al., 2014; Simon et al., 2015; Pires et al., 2016; Stylianou et al., 2019).

In sum, these data support the claim that AFM provides more precise and high-resolution images enabling study of morphological changes in eosinophils much more reliably than by optical microscopy, enabling observation of smaller changes, and improving the sensitivity to detect eosinophil alterations. This may allow for a better understanding of eosinophil morphological alterations in several diseases, in which the eosinophils are involved, such as allergic and infectious diseases, and cancer, among others.

## Data Availability Statement

All datasets generated for this study are included in the manuscript/supplementary files.

## Ethics Statement

Blood samples were obtained from peripheral blood from 3 asthmatic individuals and 3 healthy controls upon approval from the Ethics Committee of the Faculty of Medicine, University of Brasília (process number 038/1).

## Author Contributions

PE and CA performed the atomic force microscopy and optical microscopy experiments. SC, MO, AV, and TB collected and prepared the samples. SK, JL, MM-J, and PE designed the experiments and discussed the data. PE, AV, SK, JL, and MM-J prepared the manuscript. All authors approved the final version of the manuscript.

## Conflict of Interest

The authors declare that the research was conducted in the absence of any commercial or financial relationships that could be construed as a potential conflict of interest.
